# Surgical informed consent in obstetric and gynecologic surgeries: experience from a comprehensive teaching hospital in Southern Ethiopia

**DOI:** 10.1186/s12910-018-0293-2

**Published:** 2018-05-24

**Authors:** Million Teshome, Zenebe Wolde, Abel Gedefaw, Mequanent Tariku, Anteneh Asefa

**Affiliations:** 1 Department of Obstetrics and Gynecology, School of Medicine, College of Medicine and Health Sciences, Hawassa University, P.O.Box 1560, Hawassa, Ethiopia; 2 School of Public Health, College of Medicine and Health Sciences, Hawassa University, P.O.Box 70, Hawassa, Ethiopia; 3 Nossal Institute for Global Health, School of Population and Global Health, The University of Melbourne, Level 5, 333 Exhibition Street, Melbourne, 3000 Australia

**Keywords:** Surgical informed consent, Obstetrics and gynecology, Clients, Counseling

## Abstract

**Background:**

Surgical Informed Consent (SIC) has long been recognized as an important component of modern medicine. The ultimate goals of SIC are to improve clients’ understanding of the intended procedure, increase client satisfaction, maintain trust between clients and health providers, and ultimately minimize litigation issues related to surgical procedures. The purpose of the current study is to assess the comprehensiveness of the SIC process for women undergoing obstetric and gynecologic surgeries.

**Methods:**

A hospital-based cross-sectional study was undertaken at Hawassa University Comprehensive Specialized Hospital (HUCSH) in November and December, 2016. A total of 230 women who underwent obstetric and/or gynecologic surgeries were interviewed immediately after their hospital discharge to assess their experience of the SIC process. Thirteen components of SIC were used based on international recommendations, including the Royal College of Surgeon’s standards of informed consent practices for surgical procedures. Descriptive summaries are presented in tables and figures.

**Results:**

Forty percent of respondents were aged between 25 and 29 years. Nearly a quarter (22.6%) had no formal education. More than half (54.3%) of respondents had undergone an emergency surgical procedure. Only 18.4% of respondents reported that the surgeon performing the operation had offered SIC, while 36.6% of respondents could not recall who had offered SIC. All except one respondent provided written consent to undergo a surgical procedure. However, 8.3% of respondents received SIC service while already on the operation table for their procedure. Only 73.9% of respondents were informed about the availability (or lack thereof) of alternative treatment options. Additionally, a majority of respondents were not informed about the type of anesthesia to be used (88.3%) and related complications (87.4%). Only 54.2% of respondents reported that they had been offered at least six of the 13 SIC components used by the investigators.

**Conclusions:**

There is gap in the provision of comprehensive and standardized pre-operative counseling for obstetric and gynecologic surgeries in the study hospital. This has a detrimental effect on the overall quality of care clients receive, specifically in terms of client expectations and information needs.

**Electronic supplementary material:**

The online version of this article (10.1186/s12910-018-0293-2) contains supplementary material, which is available to authorized users.

## Background

Surgical Informed Consent (SIC) has long been recognized as an important component of modern medicine. SIC is an integral part of any surgical procedure and should be based on proper communication between clients and health care providers which is built on the fundamental principles of human rights, autonomy, and the right to information [1–4]. Furthermore, SIC is a process that should be integral to the continuum of care, from admission to discharge from health facility [5]. Adequate provision of SIC increases the likelihood that the client will understand the planned procedure and possible complications, and also improves client satisfaction [3]. Additional goals of SIC are to maintain trust between clients and health providers, and ultimately minimize litigation issues related to surgical procedures [6].

SIC dictates that before any kind of surgical intervention, a client should be in full agreement with the proposed procedure if he/she is in a proper frame of mind and eligible to give consent; if not a legal surrogate or guardian should sign the consent. A primary component of the SIC process is to deliver information that is adequate, timely, clear, and pertinent [7, 8], at a language level that is understandable to the client [9]. Another component of SIC is voluntarism and consent – agreeing to undergo the procedure based on information regarding benefits and risks, alternatives, and the consequences of non-treatment [1, 3]. The decision to undergo a surgical procedure should be accompanied by signature of the client or other responsible person [2, 3, 8, 10, 11].

SIC is the right of a client and the obligation of the health system; however, it is improperly performed and violated in various circumstances [12, 13]. Proper SIC can suffer due to health facility factors like lack of a standard consent form, lack of readiness to deal with urgent medical conditions, lack of health care provider awareness and experience with SIC, and heavy work load for health care providers [7, 13]. Client characteristics like younger age and low literacy level are also associated with poor quality of SIC process [11, 13, 14].

Studies of SIC validity and quality in adult clients have revealed a scarcity of data regarding contents and delivery of information during the consent process, and comprehension of information exchanged during the SIC interview [13, 15]. Current literature reports a clear need for future research to better understand the limitations of contemporary SIC processes and provide recommendations necessary to optimize the exchange and understanding of information provided during SIC [9]. This is also true in Ethiopia, where there have been no prior studies on the implementation of SIC in gynecology and obstetric procedures, either specific or general. Therefore, the purpose of this study is to assess current practice of the recommended components of standard SIC process in HUCSH among clients who have undergone obstetric and gynecologic surgeries. We believe that this study provides information on the status of SIC process in a teaching hospital that may also reflect the status of SIC in other teaching hospitals in the country and other low-income settings. The information generated will hopefully help for evidence-based decisions targeting the improvement of informed decision making for obstetric and gynecologic surgeries. The Ethiopian health sector transformation plan (2015/16–2019/20) also clearly articulates the need for evidence-based actions to improve the quality of surgical care services in health facilities [16].

## Methods

### Study design and setting

This cross-sectional study was conducted at the HUCSH Department of Obstetrics and Gynecology (Ob-Gyn) during November and December of 2016. HUCSH has a bed capacity of 400 and renders tertiary care services to a catchment population from the Southern Nations Nationalities and Peoples Region (SNNPR) and other neighboring catchments of the Oromia region. At the time of the study, the HUCSH Department of Ob-Gyn employed eight obstetrician-gynecologists, 12 resident physicians, 39 midwives, and 20 ward nurses. Between 20 and 25 practicing medical interns rotate through the department at a time. The hospital contains three operation theaters for obstetric and gynecologic surgeries. An average of about 4000 deliveries take place in the hospital each year—between 30 and 35% by cesarean section. During the study period, 648 deliveries and 350 obstetric or gynecologic surgeries were performed in the hospital.

SIC is usually conducted in the labor ward, Ob-Gyn wards, or emergency Ob-Gyn outpatient department, depending on the indication, timing, and urgency of the client’s condition. The department uses a single consent sheet for both obstetric and gynecologic surgical procedures. An Additional file 1 shows the consent form that was being used during the study. The content of the consent form originally written in local language (Amharic) is translated as “I the undersigned client (Miss/Ms _______________ have been told by the health professional that due to the condition I have I should deliver my baby by operation. I have been informed that if any complication arises as a result of the surgery I shall take full responsibility of the outcome, the hospital or the physician involved are not accountable to any possible bad outcome related to my surgery. Name and signature of the client _________________” (See Additional file 1). However, there is no clear protocol regarding who should be responsible for getting informed consent, when and where SIC should be conducted, and the best methods of delivering information.

### Study participants

Participants in this study were women who underwent elective or emergency obstetric or gynecologic surgeries during November and December of 2016. Women who had repeated surgeries during the same admission were excluded from the study to prevent confounding between the SIC processes they experienced for different surgeries. Women under the age of 18 years were ineligible to participate in the study.

### Sample size and sampling

Two hundred and thirty-nine women were invited to participate based on a single population proportion formula incorporating a 95% confidence level, 5% expected margin of error, 10% expected non-response rate, and the assumption that 17% of women would not give consent to undergo a surgery. The latter proportion was borrowed from a study conducted in a Ugandan teaching hospital, due to a lack of evidence from the Ethiopian context) [12]. Ultimately, 230 women agreed to participate (96% response rate). Women were recruited as they became eligible (i.e. following surgical procedures) and interviewed prior to discharge.

### Variables

Thirteen components of SIC were used based on international recommendations, including the Royal College of Surgeon’s standards of informed consent practices for surgical procedures. The outcome of interest was defined as receipt of at least six of these components. Women’s satisfaction with the SIC process was a secondary outcome of interest. Socio-demographic variables, service-related characteristics, and clients’ perceptions of SIC served as independent variables in this study.

### Data collection and processing

Data was collected by female nurses who had no affiliation with the study hospital, using an interviewer-administered questionnaire. The questionnaire included three main sections regarding sociodemographic characteristics, service related characteristics, and essential components of the SIC procedure. Recommendations of the Royal College of Surgeons [17] and the Ethiopian Hospital Reform Implementation Guideline [18] were used as a start-up guide to develop components of SIC assessment questions. A questionnaire prepared in English was translated to Amharic (the most commonly spoken local language). The Amharic version was translated back to English to maintain consistency, while the Amharic version was used to interview women. Interviews were conducted immediately after discharge to minimize risk of desirable responses that may happen due to clients’ concerns about their current care. Women were recruited and interviewed continuously until an appropriate sample size was achieved.

### Data quality assurance

Prior to data collection, 2 days of training was provided to all data collectors and a supervisor. Following that, a pilot test was conducted among 20 women who had obstetric or gynecologic surgical procedures at HUCSH and the interview questionnaire was modified based on their feedback. Completed questionnaires were checked for completeness and consistency on a daily basis throughout the data collection period.

### Data analysis and interpretation

Data were entered, cleaned, and analyzed using SPSS version 19 statistical package for windows. Frequency measures were calculated to describe categorical variables, whereas means or medians were computed for continuous variables after checking for normality of distributions using Kolmogorov-Smirnov and Shapiro-Wilk tests. Thirteen indicators measuring provision of informed consent for surgical procedures were used to identify the proportion of women who received the minimum essential components of SIC (Table 1). Possible responses included, “Yes,” “No,” and, “I don’t remember.” In this study, women who reported having received at least six of the 13 components of informed consent were regarded as having received the minimum acceptable level of SIC.

**Table 1:** Essential components of surgical informed consent received by respondents, Hawassa, 2016

Essential components of surgical informed consent	Response, n (%)		
	Yes	No	Do not remember
Respondent/respondent’s family was requested for an informed consent (*n* = 230)	229 (99.6)	1 (0.4)	–
Respondent/respondent’s family signed on an informed consent form (*n* = 230)	229 (99.6)	1 (0.4)	–
Respondent was informed why the surgery will be performed (indication of surgery) (*n* = 230)	200 (87.0)	30 (13.0)	–
Respondent was informed the expected time the surgery will take (*n* = 230)	33 (14.3)	194 (84.3)	3 (1.3)
Respondent was informed about presence/absence of alternative treatment option/s (*n* = 230)	56 (24.3)	170 (73.9)	4 (1.7)
Respondent was informed about type of anesthesia to be used (*n* = 230)	26 (11.3)	203 (88.3)	1 (0.4)
Respondent was given counseling aids which assist in decision making (*n* = 230)	3 (1.3)	227 (98.7)	–
Respondent was informed about potential complication/s which may arise (*n* = 230)	27 (11.7)	201 (87.4)	2 (0.9)
Respondent was informed about consequences of refusing the proposed surgery (*n* = 230)	111 (48.3)	115 (50.0)	4 (1.7)
There was a favorable environment to say “No” to the proposed surgery (*n* = 229)	15 (6.6)	214 (93.4)	–
Respondent was given adequate time for decision to sign on the informed consent form (*n* = 217)	67 (30.9)	150 (69.1)	–
Respondent was given an opportunity to ask question (*n* = 230)	186 (80.9)	44 (19.1)	–
Respondent given opportunity to choose from anesthesia options (*n* = 230)	14 (6.1)	216 (93.9)	–

The main dependent variable (receipt of minimum acceptable level of SIC) was generated by counting the total number of “Yes” responses among the 13 indicators for each woman in the study. The cut-off point (six affirmative responses) was based upon the distribution of “Yes” values and the authors’ expertise. A binary logistic regression analysis was conducted to check for an association between the sociodemographic and service related characteristics of clients and the dependent variable; odds ratio with their corresponding 95% confidence intervals are reported. All variables included in the bivariate analysis were included in the multivariate analysis. Additionally, a multinomial logistic regression analysis was done to assess whether there is a difference between the timing of SIC counseling (categorized as: the day before date of surgery, on the day of surgery, immediately before surgery, and on the operation table) between clients who had an emergency and an elective surgery.

## Results

### Socio-demographic characteristics

Forty percent of respondents were aged between 25 and 29 years (M = 28.2; SD = 7.9). Nearly a quarter (22.6%) had no formal education, while 20.9% have attended only primary school. Nearly all (92.2%) of the women involved in this study were married and more than half (53%) were housewives. The average monthly income of the respondents was 3690.7 Birr (Table 2).

**Table 2:** Sociodemographic and economic characteristics of respondents, Hawassa, 2016

Variables	Frequency (%)
Age in completed years (*n* = 229)	
15–19	4 (1.7)
20–24	62 (27.0)
25–29	92 (40.0)
30–34	33 (14.3)
35–39	20 (8.7)
40–45	11 (4.8)
46 and above	7 (3.0)
Total	229 (100.0)
Mean ± SD	28.2 ± 7.9
Educational level (*n* = 230)	
No formal education	52 (22.6)
Some primary education	48 (20.9)
Completed grade 8	18 (7.8)
Some secondary education	25 (10.9)
Completed grade 12	23 (10.0)
College and above	32 (16.4)
Total	230 (100.0)
Marital status (*n* = 230)	
Single	7 (3.0)
Married	212 (92.2)
Separated	5 (2.2)
Divorced	1 (0.4)
Widowed	5 (2.2)
Total	230 (100.0)
Religion (*n* = 230)	
Christian Orthodox	61 (26.5)
Christian Protestant	90 (39.1)
Muslim	74 (32.2)
Others	5 (2.2)
Total	230 (100.0)
Ethnicity (*n* = 230)	
Sidama	43 (18.7)
Oromo	85 (37.0)
Amhara	28 (12.2)
Gurage	29 (12.6)
Wolayita	20 (8.7)
Others	25 (10.9)
Total	230 (100.0)
Occupation (*n* = 230)	
Housewife	122 (53.0)
Private employee	13 (5.7)
Government employee	38 (16.5)
Private business	39 (17.0)
Farmer	16 (7.0)
Others	2 (0.9)
Total	230 (100.0)
Respondent has regular monthly income (*n* = 228)^a^	
Yes	166 (72.8)
< 845 Birr	17 (10.2)
≥ 845 Birr	149 (89.8)
Mean ± SD	3690.7 ± 4343.6 Birr
No	62 (27.2)
Total	228 (100.0)

^a^1USD = 23.5 Eth birr during the study period on average

### Basic service characteristics

A majority of the women (70.4%) involved in this study were referred from other health facilities. More than one third (36.6%) of respondents do not know the role of the person who conducted the SIC counseling session. The remaining participants reported to have received SIC counseling from resident physicians (27.3%), nurse-midwives (21.3%), or obstetrician-gynecologists (18.4%) (Table 3).

**Table 3:** Service related characteristics of respondents

Variables	Frequency (%)
Referred from other health facility (*n* = 230)	
Yes	162 (70.4)
No	68 (29.6)
Total	230 (100.0)
Profession of the person who gave counseling (*n* = 216)	
Obstetrician-gynecologist	32 (14.8)
Resident physician	59 (27.3)
Nurse-midwife	46 (21.3)
Did not know	79 (36.6)
Total	216 (100.0)
Schedule of obstetric/gynaecologic surgery performed (*n* = 230)	
Elective	105 (45.7)
Emergency	125 (54.3)
Total	230 (100.0)
Type of anaesthesia received (*n* = 230)	
General	49 (21.3)
Spinal	181 (78.7)
Total	230 (100.0)
Timing of counseling for informed consent (*n* = 216)	
The day before date of surgery	26 (12.0)
On the day of surgery	19 (8.8)
Immediately before surgery	152 (70.4)
On the operation table	19 (8.8)
Total	216 (100.0)

A slight majority of study participants (54.3%) had undergone emergency surgical procedure, while the rest received elective procedures. A majority (78.3%) of these procedures were performed under spinal anesthesia, while the remaining surgeries were performed under general anesthesia. More than two thirds (70.4%) of women reported to have received SIC counseling immediately prior to their surgery (before client was put on the operation table), while 8.8% (2% among elective surgical clients and 14.8% among emergency surgical clients; *p* < 0.001) reported to have received counseling on the operation table. Meanwhile, 12% of women reported that they had received counseling 1 day prior to their surgery and 8.8% reported to have received counseling on the same day (Table 3). The odds of receiving SIC on the operation table rather than receiving it 1 day prior to the date of surgery was lower among clients who had an elective surgery than those who had an emergency surgery (OR: 0.02; 95% CI: 0.01–0.10).

### Components of surgical informed consent received

Almost all (99.6%) of the respondents or their family members were asked to provide written consent, and all agreed. Most women (87%) reported that they received information about the indication(s) for undergoing the surgical procedure. Only 14.3% of the respondents were informed about the expected duration of their surgery. Nearly three quarters (73.9%) of women were not informed about possible alternatives to surgical intervention; 71.4% among elective and 76% among emergency clients (*p* = 0.09). Few (11.3%) respondents were informed about the type of the anesthesia to be administered. Only 3 (1.3%) women reported being given counseling aids to help them make decisions about their surgery; receipt of counseling aids did not vary with clients’ educational status (*p* = 0.30). Most (87.4%) respondents did not receive any information concerning possible complications related to their surgery. Half (50%) of the women involved in this study were not informed about the possible consequences if they chose not to have the surgical procedure. Only 14 (6.1%) were given the opportunity to choose from the available anesthesia options (general or spinal) (Table 1).

### Receipt of the minimum recommended components of surgical informed consent

Upon counting the total numbers of the components of SIC respondents received before their surgery, 26.5% of the respondents were identified to have received at least six of the 13 components of SIC suggested by the investigators (13.9% among housewives, *p* < 0.001). Furthermore, there was no significant difference in the number of SIC components received between clients who had emergency (23.2%) and elective (30.5%) surgeries (*p* = 0.21). The mean number of components of SIC received by respondents was computed to be 4.57 with a standard deviation of 1.95. The multivariate logistic regression analysis revealed that, none of the sociodemographic and service related characteristics of clients, except the timing of counseling, were found to be associated with receipt of the minimum (at least six) components of SIC (Table 4).

**Table 4:** Factors associated with receipt of the components of surgical informed consent, Hawassa, 2016

Variables	Received at least six SIC components		Crude odds ratio (95% CI)	Adjusted odds ratio (95%CI)
	Yes	No		
Age in completed years				
15–24	13	53	Ref.	Ref.
25–34	39	86	1.8 (0.9, 3.8)	1.1 (0.4, 3.1)
35–44	7	19	1.5 (0.5, 4.3)	1.8 (0.4, 8.4)
45 and above	2	10	0.8 (0.2, 4.2)	0.2 (0.1, 4.6)
Educational level				
No formal education	7	45	Ref.	Ref.
Some primary education	12	36	2.1 (0.8, 6.0)	2.4 (0.6, 10.4)
Completed grade 8	2	16	0.8 (0.2, 4.3)	0.9 (0.1, 7.8)
Some secondary education	2	23	0.6 (0.1, 2.9)	0.4 (0.1, 3.4)
Completed grade 12	3	20	1.0 (0.2, 4.1)	0.7 (0.1, 4.5)
College and above	35	29	7.8 (3.0, 19.8)	1.8 (0.3, 10.5)
Marital status				
Single	0	7	Ref.	Ref.
Married	61	151	–	–
Separated	0	5	–	–
Divorced	0	1	–	–
Widowed	0	5	–	–
Religion				
Christian Protestant	27	63	Ref.	Ref.
Christian Orthodox	23	38	1.4 (0.7, 2.8)	1.2 (0.5, 3.2)
Muslim	10	64	0.4 (0.2, 0.8)	0.4 (0.1, 1.3)
Others	1	4	0.6 (0.1, 5.5)	0.4 (0.1, 13.0)
Occupation				
Housewife	17	105	Ref.	Ref.
Farmer	4	12	2.1 (0.6, 7.1)	1.2 (0.2, 6.9)
Private employee	7	6	7.2 (2.1, 24.0)	1.3 (0.2, 7.7)
Government employee	19	19	6.2 (2.7, 14.0)	1.2 (0.3, 5.7)
Private business	14	25	3.5 (1.5, 7.9)	1.1 (0.3, 3.8)
Monthly income				
< 845 Birr	2	15	Ref.	Ref.
≥ 845 Birr	57	92	4.5 (1.0, 21.1)	3.9 (0.6, 25.8)
Referred from other health facility				
Yes	30	132	Ref.	Ref.
No	31	37	3.7 (2.0, 6.9)	2.3 (0.9, 5.8)
Schedule of surgery				
Elective	32	73	Ref.	Ref.
Emergency	29	96	0.7 (0.4, 1.2)	1.7 (0.7, 4.0)
Timing of counseling for informed consent				
The day before date of surgery	11	15	Ref.	Ref.
On the day of surgery	9	10	1.2 (0.4, 4.0)	0.2 (0.1, 1.3)
Immediately before surgery	41	111	0.5 (0.2, 1.2)	0.1 (0.02, 0.6)
On the operation table	0	19	–	–

### Satisfaction with surgical informed consent process

**Fig. 1:**
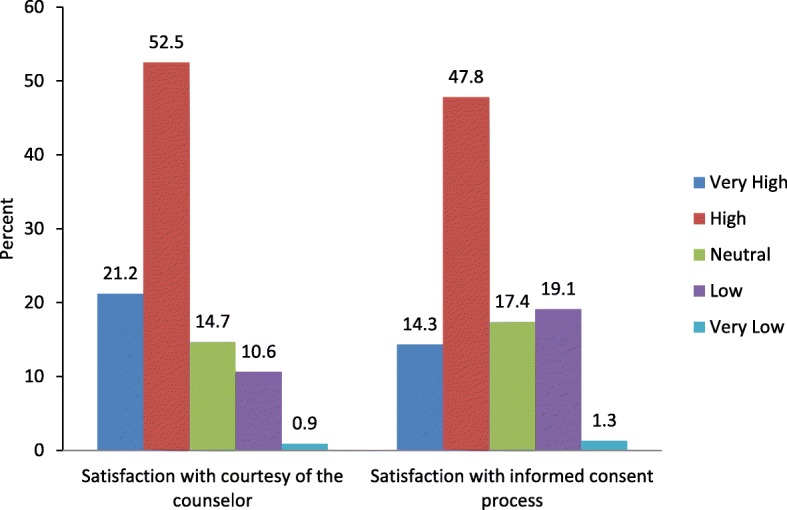
Respondents’ level of satisfaction with surgical informed consent process, Hawassa, 2016

Women were asked to assess their level of satisfaction with the SIC service they received prior to their surgical procedure on a five-point scale. Nearly two thirds (62.1%) of women reported that they were very satisfied (14.8%) or satisfied (47.3%) with the service, while 19.1% were dissatisfied, and 1.3% were very dissatisfied. Majority of women were either very satisfied (21.2%) or satisfied (52.5%) with the courtesy of the SIC provider (Fig. 1).

## Discussion

This study reports on women’s experiences of SIC in a tertiary teaching hospital in Hawassa, Ethiopia. Our focus on obstetric and gynecologic surgeries is due to the fact that there are reports of malpractice in surgical care of obstetric and gynecologic clients, which is believed to affect informed decision making by clients, in Ethiopia which warrants further exploration [19].

This study reveals that a significant proportion (73.5%) of women did not receive at least six of the 12 components of SIC (the minimum recommendation). The absence of association between respondents’ sociodemographic characteristics and whether the surgery is emergency or elective with receipt of the minimum recommendation suggests that the SIC rendered by health professionals do not vary by clients’ attributes. Almost all respondents or their families provided written consent to undergo a surgical procedure, indicating that written consent is obtained as a routine practice to fulfill a requirement, and not as part of the recommended SIC practices. When performed optimally, the informed consent process creates an opportunity for the client and/or family to ask questions and clarify concerns, and at the same time provides an opportunity for health care providers to gain trust and build rapport with clients as well as families [6, 17, 20, 21]. In this study, 80.9% of the respondents were given the chance to ask questions during the SIC process. However, counseling aids that inform clients about surgical procedures and play a significant role in informed decision-making were provided to almost none (1.3%) of the respondents. Ideally, SIC should support the fundamental principles of client autonomy and self-determination with the aims of protecting clients and health care providers [6, 21].

According to recent guidelines, the individual who obtains consent from a client should be the same person who performs the procedure, or someone specially trained to counsel clients about the procedure and related issues [17]. The person who provides surgical care should be well versed in the surgical skills, possible complications, and risks associated with the particular procedure, as well as alternative treatment options for the client, if any. However, only 21.3% of participants in this study received the essential components of SIC from nurses or midwives who were neither involved in the surgical procedure, nor had any special training in client counseling regarding SIC. This finding is more favorable than a similar report from Pakistan, where consent was obtained by nurses who were uninvolved in surgical procedures and untrained in SIC counseling 60% of the time [22]. However, in high income countries, consent for surgical procedures is most often obtained by senior doctors responsible for performing a surgery [23].

For a consent process to be considered valid, it should not be conducted under pressure. Hence, consent should not be obtained on the day of the surgery, putting clients under significant pressure to make a decision. The time offered for individual clients to provide consent after the receipt of all relevant information may vary based on the schedule of the procedure [24]. In this study, informed consent was most often obtained either immediately prior to the surgery (70.4%) or after the client was already on the operation table (8.8%). It is not ethically blameworthy that SIC was provided on the operation table for clients undergoing emergency surgeries. However, almost equal proportions of elective (68.3%) and emergency (72.2%) surgical clients received SIC immediately prior to their surgery, which would have been earlier for elective surgical clients. This implies that informed consent in the study hospital mainly focused on signed consent forms rather than informed decisions. On a similar note, provision of information does not guarantee shared understanding. Clients in this study belonged to various ethnic groups that speak different languages; however, Amharic is the only working language in the hospital. Counseling aids prepared in various languages and consistent availability of multilingual counsellors would mitigate these challenges.

In this study, participants were not: informed of the indication(s) of their surgeries (13%), often given the opportunity to ask questions (19.1%), informed about alternative treatment options (73.9%), or counseled concerning the possible complications of the procedure (87.4%). Similarly, a study conducted in a Ugandan teaching hospital found that participants did not receive information on the type of the surgery they would have (17%), and did not have all of their questions answered (43.9%) [12]. Along the same lines, a study from Pakistan revealed that participants had no information on the details of their surgery (91.1%), were not informed about the possible complications (96.6%), and were not given the chance to ask questions (60.6%) [22]. Differences among the three studies may be due to varying SIC practices, service delivery arrangements, or resource capacities.

Most clients received counseling from nurses-midwives and junior physicians who lacked special training in counseling. Only 14.8% of participants received counseling from the senior physicians performing their surgeries. Clients’ perceptions and understanding of information can be influenced by factors like the health care provider’s duration of training, age, gender, and years of experience [6, 11, 13]. Content of the consent form, if properly designed, also aids in proper transfer of information and serves as a tool to assist a client in informed decision-making. The consent document should address all information relevant to an informed decision, as this information affects the quality of informed consent [17].

A signed consent form is only valid when there is adequate transfer of information and only confirms that the client has agreed to the next stage of treatment. This is by no means sufficient evidence in the court of law, which is why some health centers have begun to use standardized audiovisual and multimedia aids for the consent process [6, 17]. Based upon the results of this study, we recommend revision of the SIC process at the study hospital to improve informed decision making prior to obstetric and gynecologic surgeries. The revision is recommended because of the missing SIC components as objectively assessed by the investigators and the rudimentary SIC form currently used in the study hospital, which is far from meeting the requirements of standard SIC counseling [6]. This is one of the very few studies conducted in Ethiopia in the immediate postoperative period. Interviewing women upon discharge minimized the chance of recall bias. Because it was conducted in a tertiary teaching hospital, it would be difficult to generalize the findings of this study to other categories of hospitals in Ethiopia. This study was also limited in terms of identifying what women didn’t learn, but what might have mattered to them, during SIC process. We recommend that future studies consider a multidimensional approach that includes observations and interviews with health care providers in different levels and types of hospitals across the country. Furthermore, inclusion of all types of clients undergoing surgical procedures and assessing clients’ expectations of information rendered during counseling would lead to a clearer understanding of SIC in Ethiopia.

## Conclusions

A majority of women who underwent an obstetric or a gynecologic surgery or both did not receive comprehensive information during the SIC process in the study hospital. This gap in the provision of comprehensive and standard pre-operative counseling for surgical procedures diminishes the overall quality of care clients receive and the ability of the health facility to meet clients’ expectations and information needs. These findings suggest the need for evidence-based improvements in the delivery of SIC.

## Additional file

Additional file 1: Consent form. A consent form that was being used for obstetric and gynecologic surgeries during the study period (translated from an Amharic language version). (DOCX 201 kb)

## Abbreviations

HUCSH: Hawassa University Comprehensive Specialized Hospital
Ob-Gyn: Obstetrics and Gynecology
SD: Standard Deviation
SIC: Surgical Informed Consent
SPSS: Statistical Package for the Social Sciences
