# Supplementary material for: Surgical informed consent in obstetric and gynecologic surgeries: experience from a comprehensive teaching hospital in Southern Ethiopia
Source: BMC Med Ethics. 2018 May 24;19:38. doi: 10.1186/s12910-018-0293-2 (PMC5968605; doi:10.1186/s12910-018-0293-2)
Supplement: Supplementary file 1 — Consent form. A consent form that was being used for obstetric and gynecologic surgeries during the study period (translated from an Amharic language version). (DOCX 201 kb) [file 12910_2018_293_MOESM1_ESM.docx]

**Consent Form - that was being used for obstetric and gynecologic surgery (translated from the Amharic version displayed below)**

Date-----------------

I the undersigned client (Miss/Ms ------------------------------ have been told by the health professional that due the condition I have I should deliver my baby by operation, I have been informed that if any complication arises as a result of the surgery I shall take full responsibility of the outcome, the hospital or the physician involved are not accountable to any possible bad outcome related to my surgery.

Name and signature of the client.........................................


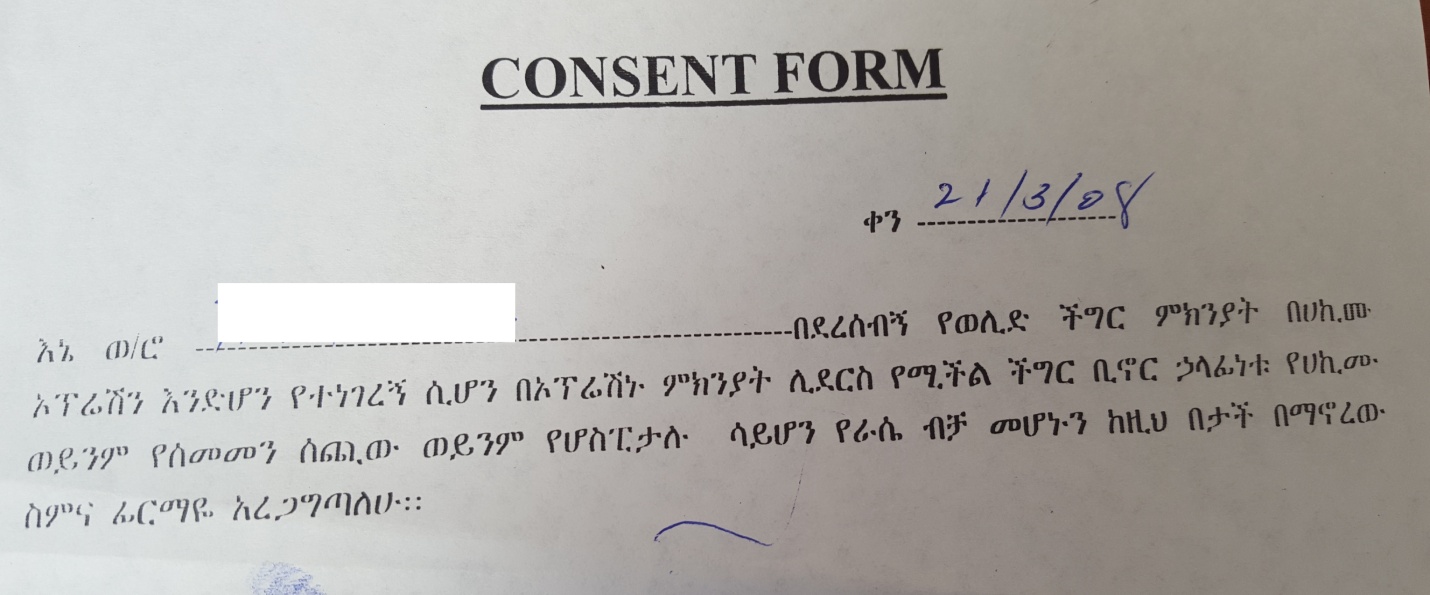


The Amharic version consent form
